# Unemployment and COVID-19-related mortality: a historical cohort study of 50,000 COVID-19 patients in Fars, Iran

**DOI:** 10.4178/epih.e2022032

**Published:** 2022-03-12

**Authors:** Alireza Mirahmadizadeh, Mohammad Taghi Badeleh Shamooshaki, Amineh Dadvar, Mohammad Javad Moradian, Mohammad Aryaie

**Affiliations:** 1 Non-Communicable Diseases Research Center, Shiraz University of Medical Sciences, Shiraz, Iran; 2 Depatment of Health Psychology, Golestan University of Medical Science, Gorgan, Iran; 3 Shiraz University of Medical Sciences, Shiraz, Iran; 4 Trauma Research Center, Shahid Rajaee (Emtiaz) Trauma Hospital, Shiraz University of Medical Sciences, Shiraz, Iran; 5 Department of Epidemiology, School of Health, Shiraz University of Medical Sciences, Shiraz, Iran

**Keywords:** Unemployment, COVID-19, Mortality, Iran

## Abstract

**OBJECTIVES:**

Previous studies have estimated the risk of death associated with unemployment in the coronavirus disease 2019 (COVID-19) pandemic, but no studies have examined unemployment before COVID-19 infection as a risk factor for COVID-19-related mortality. Thus, this study aimed to investigate COVID-19 mortality among this population.

**METHODS:**

Data on 50,038 people aged 25-59 years were collected from 38 agencies in Fars Province, Iran, from February 2020 to July 2021. Follow-up lasted from participants’ diagnosis with COVID-19 based on the results of a reverse transcription-polymerase chain reaction test to participants’ death or the end of the study period. The association between unemployment and COVID-19-related mortality was estimated using the Poisson regression method, and a sensitivity analysis was conducted to calculate the E-value.

**RESULTS:**

Unemployment was associated with a 2.41-fold (95% confidence interval [CI], 2.01 to 2.90) higher age-adjusted and sex-adjusted risk of COVID-19-related mortality. The adjusted Poisson regression analysis showed 8.82 (95% CI, 6.42 to 12.11), 2.84 (95% CI, 1.90 to 4.24), and 1.58 (95% CI, 1.24 to 2.01) times higher risks of COVID-19-related mortality among unemployed people aged 25-39 years, 40-49 years, and 50-59 years, respectively, than among their employed counterparts. Unemployment increased the risk of COVID-19 mortality by 3.31 (95% CI, 2.31 to 4.74) and 2.30 (95% CI, 1.86 to 2.84) times in female and male, respectively. The E-value was 3.43, reflecting the minimum strength of confounding required to shift the association between unemployment and COVID-19-related mortality toward the null.

**CONCLUSIONS:**

Unemployment prior to COVID-19 infection increased the risk of COVID-19-related mortality. COVID-19-related mortality disproportionately impacted unemployed women and younger unemployed people.

## INTRODUCTION

Approximately 35 million coronavirus disease 2019 (COVID-19) infections have been recorded worldwide, resulting in more than 610,000 estimated deaths by August 2021 [1]. COVID-19 may disproportionately affect unemployed people due to economic distress and the deferment of care [2,3], leading to an excess in mortality. Several recent studies have shown that deaths attributed to COVID-19-related unemployment have disproportionately varied by sex, age, race and ethnicity, and education level [3-5]. These studies have suggested that different policy decisions during the COVID-19 pandemic, including universal masking, community-wide lockdowns, social distancing, travel restrictions, quarantine, stay-at-home orders, and contact tracing, were effective tools for reducing COVID-19 transmission and mortality [6-8], but also cause a decline in economic growth [9,10], which substantially impacted health and human well-being [11,12].

Despite previous studies estimating the risk of death associated with unemployment related to the COVID-19 pandemic [3-5], no studies have examined COVID-19-related mortality among those who were unemployed prior to COVID-19 infection. Moreover, although the association between unemployment, poor health, and increased mortality seems robust [13,14], whether the association between unemployment and mortality is causal remains an open question. Chronic diseases could be mediating or confounding variables in the association between unemployment and mortality, meaning that chronic diseases could be caused by adverse behavioral changes related to unemployment or could be a shared cause of both unemployment and mortality.

In the current study, we aimed to address 2 questions. First, given that prior studies have observed an association between unemployment (or losing one’s job as a result of the COVID-19 pandemic) and mortality, we examined whether those who were unemployed before the COVID-19 pandemic (rather than as a result of the COVID-19 pandemic) and were infected with COVID-19 had higher mortality than that of employed people. Second, we assessed the predicted associations among subgroups of the study participants based on their chronic disease status since chronic diseases may have been mediating or confounding variables.

## MATERIALS AND METHODS

### Data and study population

For this study, data were collected from 38 state agencies and combined for research purposes at a medical care monitoring center (MCMC) storage facility in Fars Province in southern Iran from February 2020 to July 2021. The MCMC is a system for reporting COVID-19 cases in hospitals according to the instructions of Iran’s health ministry. Using this system, data on admission, diagnosis, treatment regimens, and follow-up for each patient are collected. In Fars and throughout Iran, data on hospitalized patients are recorded using a health information system and paper-based medical records (details on the MCMC system, its data quality, and its quality assurance can be found in a study by Zarei et al. [15]). Individuals aged 25-59 years were included in the study to account for nearly all working-age people. The follow-up period began at the time of diagnosis with COVID-19 based on a reverse transcription-polymerase chain reaction test or computed tomography scan findings and ended either when the subject died or when the study period ended. A small percentage (0.01%) of individuals were excluded due to a lack of information on their employment status. A total of 50,038 subjects were ultimately included in the final study population.

### Exposure, potential mediating or confounding variables, and outcomes

The study population was categorized into 2 groups (employed and unemployed) according to their exposure status, and COVID-19-related mortality was defined as the outcome. Unemployment was defined as having no income for at least a year preceding the COVID-19 pandemic. Moreover, demographic data, data on sex and age, and data on comorbid diseases (coded as 1= yes and 0= no for each chronic disease), including on diabetes, cancer, cardiovascular disease, pulmonary disease, chronic kidney disease, and HIV, were also collected by the MCMC system.

### Statistical analysis

Descriptive statistics were used to define the categorical variables, and risk ratios and 95% confidence intervals (CIs) were obtained using the Poisson regression method with a robust standard error. All analyses were performed using Stata version 14 (StataCorp., College Station, TX, USA). Using the multiple Poisson regression method, we estimated the age-adjusted and sex-adjusted risk ratios for the association between unemployment and COVID-19-related mortality. We then repeated this procedure for each chronic disease, since they functioned as effect modifiers or mediating factors rather than as confounding variables concerning the association of interest in this study. Since the outcome of interest was binary, we used log-Poisson regression models to estimate valid risk ratios in addition to robust standard errors to correct the related standard errors obtained from the Poisson models (a detailed explanation of the log-Poisson regression model and robust standard error for binary outcomes can be found in a study by Mansournia et al. [16]) as follows:


$$\log\left(\pi \right)=\beta_{0}+\beta_{\mathrm{unemployment}}+\beta_{\mathrm{age}}+\beta_{\mathrm{sex}}$$

In which β is the coefficient of the predictor variables and log(π) is the logarithm of the risk.

### Sensitivity analysis

We computed the E-value, which indicates the minimum strength of unknown or unmeasured confounding variables needed to fully explain the association between unemployment and COVID-19-related mortality beyond the measured confounders on the risk ratio scale [17], as follows:


E-value= risk ratio+square root {risk ratio×(risk ratio−1)} 

### Ethics statement

The protocol of this study was reviewed and approved by the Ethics Committee of Shiraz University of Medical Sciences (code: IR.SUMS.REC.1399.1165).

## RESULTS

Out of the 50,038 participants aged 25-64 years, 1,838 (3.7%) were unemployed. Of the employed participants, 938 (1.9%) died, while 148 (8.0%) of the unemployed subjects died. The demographic characteristics and medical histories of participants in relation to COVID-19 deaths are summarized in Table 1.

The age-sex-adjusted Poisson regression indicated that unemployed participants had a 2.41 (95% CI, 2.01 to 2.90) times higher mortality risk than those who were employed. The age-adjusted risk ratio of mortality was 3.31 (95% CI, 2.31 to 4.74) for unemployed female compared to employed female and 2.30 (95% CI, 1.86 to 2.84) for unemployed male compared to employed male. For those aged 25-39 years, 40-49 years, and 50-59 years, the risk ratios of mortality were 8.82 (95% CI, 6.42 to 12.11), 2.84 (95% CI, 1.90 to 4.24), and 1.58 (95% CI, 1.24 to 2.01), respectively, for unemployed participants compared to employed participants in the same age groups. This study showed that unemployment increased the risk of COVID-19-related mortality in nearly all of the subgroups under investigation. The risk ratios for the association between employment status and COVID-19-related mortality according to chronic disease subgroups are shown in Table 2. The sensitivity analysis indicated that an E-value of at least 3.43 was required for the adjusted risk ratio of 2.41 to shift the association between unemployment and COVID-19-related mortality toward the null.

## DISCUSSION

This study examined the association between unemployment and COVID-19-related mortality among working-age people in the province of Fars in southern Iran. Our findings demonstrated that, although the risk of COVID-19-related mortality increased with age, it was substantially higher among younger unemployed participants than among younger employed participants in the corresponding age groups than older unemployed participants compared to older employed participants. Older unemployed people may tend to have more financial resources and savings when they lose a job, making younger unemployed people more vulnerable to financial stress, which can lead to deferred care and increase their risk of COVID-19-related mortality. Future studies should examine this phenomenon further.

The results of the present study showed that unemployed female were more likely to die from COViD-19 infection than employed female, and the magnitude of this difference was greater than that between unemployed and employed male. Due to socioeconomic and cultural factors in Iran [18,19], females are more likely to have part-time jobs and lower wages than males; as a consequence, job loss may pose more economic distress for female, which would make it harder for them to afford the cost of COVID-19 treatment. The long-term effect of COVID-19-related job loss on mortality seems to be worse for female than for male [5], whereas the opposite findings have been observed related to the short-term effect [3]. A cohort study conducted by d’Errico et al. [20] found an unemployment-mortality association in males, while no association was detected in females. Although a few studies have investigated mortality associated with COVID-19-related unemployment [3-5], no previous studies have examined COVID-19-related mortality among those who were unemployed prior to COVID-19 infection.

The present study found that unemployment increased the risk of COVID-19-related mortality in nearly all of the chronic disease subgroups, but the strength of the association varied according to the chronic disease, which suggests that chronic diseases functioned as effect modifiers or mediating factors rather than as confounding variables concerning the association of interest in this study. A robust literature analysis found that the association between unemployment and mortality was most likely a causal relationship [14,21]. Therefore, unemployment may be a proxy for other factors such as low socioeconomic status, poor nutrition, and weak social support that affect COVID-19-related outcomes, including mortality, even though healthcare services related to COVID-19 are free of cost according to public health policy in Iran.

There were several limitations to our study. First, unemployment is associated with other factors related to high mortality, such as economic distress. Therefore, the lack of data on the participants’ precise income level and health insurance status may have distorted the results of our study. Well-designed cohort studies have observed a consistent poverty-mortality relationship, with each extra US$10,000 of annual income potentially reducing mortality by over 50% [22]. Second, no information on the participants’ education level, family size, duration of unemployment, social support system, and amount in savings was included in the MCMC cohort data, and further studies are needed to account for the effects of these variables on the association between unemployment and COVID-19-related mortality. Nonetheless, the sensitivity analysis yielded an E-value of 3.43, indicating the magnitude of confounding that would be required to shift the association between unemployment and COVID-19-related mortality toward the null.

In conclusion, this study adds new insights to the existing body of work on this topic. Unemployment prior to COVID-19 infection was found to increase the risk of COVID-19-related mortality. COVID-19-related mortality also disproportionately burdened unemployed female and younger unemployed people.

## Figures and Tables

**Table 1.** Demographic characteristics, medical history, and risk ratio in relation to coronavirus disease 2019 deaths in 50,038 participants

Characteristics	Subjects, n	Death, n (%)	Crude	Adjusted
Entire cohort	50,038	1,086 (2.2)		-
Sex				
Female	27,150	476 (1.7)	1.00 (reference)	-
Male	22,888	610 (2.7)	1.52 (1.35, 1.71)	-
Age (yr)				
25-39	29,227	268 (0.9)	1.00 (reference)	-
40-49	11,913	275 (2.3)	2.51 (2.13, 2.97)	-
50-59	8,898	543 (6.1)	6.65 (5.76, 7.68)	-
Employment status				
Employed	48,200	938 (1.9)	1.00 (reference)	1.00 (reference)
Unemployed	1,838	148 (8.0)	4.13 (3.50, 4.88)	2.41 (2.01, 2.90)
Chronic disease status				
No chronic diseases	42,705	564 (1.3)	1.00 (reference)	1.00 (reference)
At least 1 chronic disease	7,333	522 (7.1)	5.38 (4.79, 5.06)	3.10 (2.68, 3.58)
Diabetes				
No	47,669	913 (1.9)	1.00 (reference)	1.00 (reference)
Yes	2,369	173 (7.3)	3.81 (3.25, 4.46)	1.84 (1.56, 2.18)
Cancer				
No	48,936	958 (2.0)	1.00 (reference)	1.00 (reference)
Yes	1,102	128 (11.6)	5.93 (4.98, 7.06)	3.96 (3.29, 4.76)
Cardiovascular disease				
No	47,107	901 (1.9)	1.00 (reference)	1.00 (reference)
Yes	2,931	185 (6.3)	3.30 (2.82, 3.84)	1.55 (1.31, 1.83)
Pulmonary disease				
No	49,194	1,037 (2.1)	1.00 (reference)	1.00 (reference)
Yes	844	49 (5.8)	2.75 (2.08, 3.63)	1.77 (1.34, 2.34)
Chronic kidney disease				
No	49,292	1.016 (2.1)	1.00 (reference)	1.00 (reference)
Yes	676	70 (9.4)	4.55 (3.61, 5.73)	2.64 (2.07, 3.35)
HIV				
No	49,572	1.066 (2.1)	1.00 (reference)	1.00 (reference)
Yes	466	20 (4.3)	1.99 (1.29, 3.07)	1.49 (0.96, 2.30)

Values are presented as risk ratio (95% confidence interval).

1 Adjusted for age and sex.

**Table 2.** Risk ratios for the association between employment status and coronavirus disease 2019 (COVID-19)-related mortality by subgroup among 50,038 participants

Variables^1^	Unemployed, n (%)	Crude	Adjusted for mortality^2^
Sex			
Female	362 (1.3)	5.51 (3.93, 7.73)	3.31 (2.31, 4.74)
Male	1,476 (6.4)	3.37 (2.77, 4.09)	2.30 (1.86, 2.84)
Age (yr)			
25-39	632 (2.2)	9.87 (7.29, 13.25)	8.82 (6.42, 12.11)
40-49	414 (3.5)	3.02 (2.05, 4.44)	2.84 (1.90, 4.24)
50-59	792 (8.9)	1.58 (1.25, 2.01)	1.58 (1.24, 2.01)
Chronic disease status			
No chronic diseases	1,209 (2.8)	3.78 (2.88, 4.95)	1.89 (1.42, 2.53)
At least 1 chronic disease	629 (8.6)	2.28 (1.84, 2.81)	2.19 (1.75, 2.73)
Diabetes			
No	1,690 (3.5)	4.47 (3.73, 5.35)	2.56 (2.10, 3.13)
Yes	148 (6.2)	1.85 (1.18, 2.89)	1.72 (1.08, 2.73)
Cancer			
No	1,685 (3.4)	4.16 (3.47, 5.00)	2.32 (1.90, 2.83)
Yes	153 (13.9)	1.43 (0.95, 2.15)	1.48 (0.95, 2.30)
Cardiovascular disease			
No	1,627 (3.4)	3.96 (3.27, 4.80)	2.23 (1.80, 2.75)
Yes	211 (7.2)	3.11 (2.22, 4.35)	2.96 (2.07, 4.25)
Pulmonary disease			
No	1,788 (3.6)	4.26 (3.59, 5.05)	2.48 (2.05, 2.99)
Yes	60 (7.1)	1.48 (0.61, 3.60)	1.18 (0.48, 2.92)
Chronic kidney disease			
No	1,750 (3.5)	4.02 (3.36, 4.80)	2.31 (1.90, 2.81)
Yes	88 (11.8)	2.39 (1.45, 3.95)	2.38 (1.42, 3.98)
HIV			
No	1,823 (3.7)	4.18 (3.54, 4.95)	2.43 (2.02, 2.93)
Yes	15 (3.2)	1.58 (0.22, 11.07)	1.33 (0.21, 8.31)

Values are presented as risk ratio (95% confidence interval).

1 For each stratum of the variables, employed participants were considered the reference group; For example, in the diabetes stratum, the adjusted risk ratios of 2.56 and 1.72 indicate that the risk of COVID-19-related mortality was 2.56 and 1.72 times higher for unemployed people than for employed people among those with and without diabetes, respectively.

2 Adjusted for age and sex except in the age and sex stratum; In the sex stratum, we estimated the risk ratio adjusted for age only, and, in the age stratum, we estimated the risk ratio adjusted for sex only.
